# A New Role of Vemurafenib as a Neoadjuvant Treatment of Axillary and Brain Melanoma Metastases

**DOI:** 10.1155/2013/794239

**Published:** 2013-12-23

**Authors:** Idit Melnik, Michal Lotem, Boris Yoffe

**Affiliations:** ^1^Department of General and Vascular Surgery, Barzilai Medical Center, Hahistadrut Street 2, 78278 Ashkelon, Israel; ^2^Center for Melanoma and Cancer Immunotherapy, Sharett Institute of Oncology, Hadassah Hebrew University Medical Center, P.O. Box 12000, 91120 Jerusalem, Israel

## Abstract

Vemurafenib is approved by the FDA for the management of unresectable or metastatic melanoma. However, its role as a neoadjuvant therapy has not been determined. We present the first documented case in which vemurafenib induced complete tumor necrosis of both lymph node and brain metastases within one month or less, an outcome that indicated that the patient was a good candidate for excisional surgery.

## 1. Introduction

BRAF is a protein kinase of the MAPK signaling pathway, which regulates cellular proliferation, survival, and invasion [1]. Mutations in the gene encoding BRAF were first described in malignant melanoma in 2002 [2]. Approximately 50% of all melanomas harbor a mutation substituting valine for leucine in position 600 of the protein [3, 4]. Nine years after the mutation was discovered, vemurafenib, a selective BRAF inhibitor, was approved by the United States Food and Drug Administration (FDA) as a first line single agent for the treatment of unresectable or metastatic malignant melanoma with a BRAF V600E mutation as detected by an FDA-approved test [5–8]. Vemurafenib treatment is associated with some benefit in the large majority of patients even when tumor shrinkage does not meet the formal requirements of an objective response. In about 50% of the patients the response can be dramatic, starting within weeks, in the process reducing tumor masses in any parenchymal organ involved [9, 10].

Initial excitement over BRAF inhibitors gave way to the realization that the response is temporary and that subsequent development of resistance and bypass mechanisms is the rule. The acquired resistance and the limited duration of response—lasting for a median of 6.9 months [11]—led to the definition of this treatment as palliative rather than curative. However, there are clinical situations in which the availability of a potent treatment for advanced, bulky melanoma tumors opens a curative window of opportunity for patients. Here we report the case of a male patient with metastatic melanoma who exhibited complete necrosis of a large tumor following treatment with vemurafenib and was therefore eligible for surgical resection to relieve severe symptoms.

## 2. Case Report

**Figure 1:**
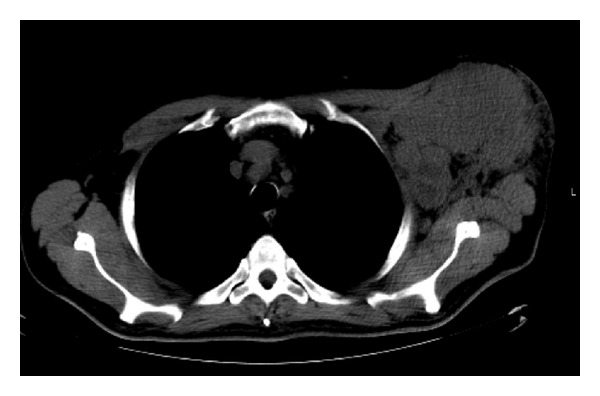
CT scan demonstrating an enlarged lymph node in left axilla with hypodense areas.

A 59-year-old Caucasian male presented to our center with a rapidly growing mass in his left axilla. He complained of weight loss, night sweating, and daily fevers. Physical examination revealed lymph node enlargement only in the left axilla, but neither loss of motor function of the left arm nor neurologic deficit presented. A full dermatological examination did not reveal any concerning lesions. Incisional biopsy of the mass demonstrated malignant melanoma metastatic to regional lymph nodes, which stained positively for S100 and MART-1. BRAF mutation status was found to be V600E using Cobas 4800. The patient underwent a total body CT with no further findings, except for the axillary mass, which was observed to be extensive, poorly circumscribed, and infiltrating adjacent structures, and therefore, it was assessed as unresectable (Figure 1). The patient was thus diagnosed with stage IIIc unresectable melanoma of unknown origin. As he was a good candidate for BRAF inhibition, the patient was treated with vemurafenib tablets, 960 mg BID. From the first week of treatment he reported an immediate improvement in his general condition and shrinkage of the painful, ulcerating axillary mass.

**Figure 2:**
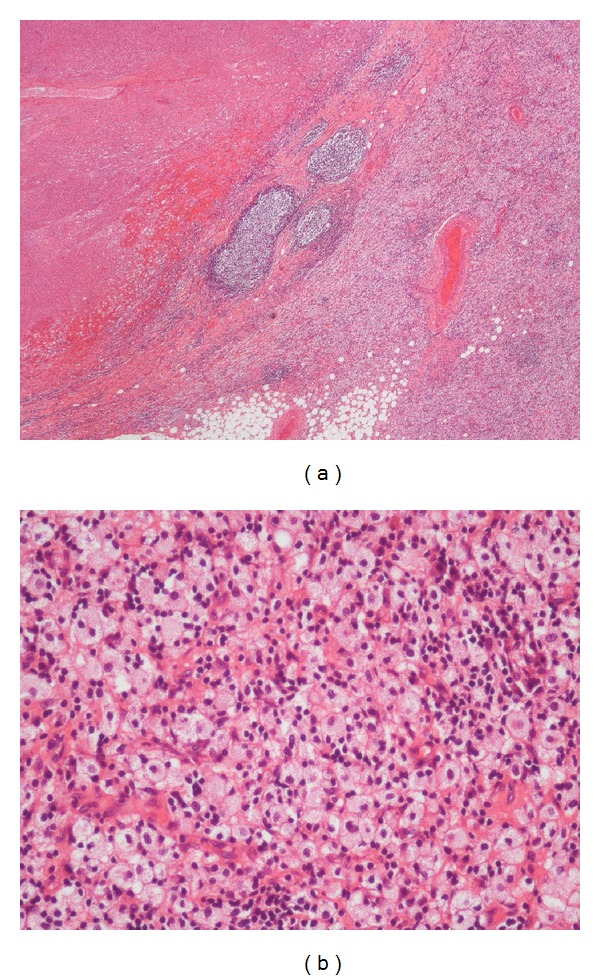
(a) Small lymphoid follicles at the edge of a necrotic area. (b) Sheets of foamy histiocytes mixed with small lymphoid cells found outside the necrotic area.

Although the initial positive results of therapy were optimistic, because such improvement has been shown to be only temporary—often followed by a state of increased resistance and progression of the disease—one month after beginning treatment we decided to operate. Surgical resection revealed a mass of 14 cm in diameter that penetrated the pectoralis major and minor muscles and encircled the axillary artery, vein, and nerve. The entire mass, including the pectoralis muscle and skin above it, was removed. The pathological examination revealed foci of a totally necrotic tumor with no observable traces of viable tissue (Figure 2).

Following his recovery, the patient returned to his homeland overseas and stopped treatment, despite medical advice to the contrary. Two months later he consented to undergo a PETCT, which revealed brain metastases and increased FDG uptake in the left axilla in a regrown mass. The patient was started immediately on vemurafenib, and in parallel, after a month his brain tumor was surgically excised. Surprisingly, despite the short time that had elapsed since treatment began (one month), the brain metastasis was already completely necrotic.

## 3. Discussion

Malignant melanoma is the most aggressive type of skin cancer. Worldwide, incidence and death rates have been rising for the last 30 years [12], and a high proportion of that increase has been younger adults [13]. Most cases are diagnosed at an early stage, a setting in which surgical excision is usually curative.

Advanced metastatic melanoma is a formidable challenge for the treating physician. A deeper understanding of the molecular pathogenesis of melanoma has revealed that in 40% to 60% of the cases, the BRAF mutation is present in the tumor cells [14, 15]. This finding has driven efforts at discovering new drugs that target the mutation [5–8].

Vemurafenib is a potent inhibitor of the kinase domain in mutant BRAF. A multicenter, phase II study (BRIM-2) evaluated vemurafenib in patients who had BRAF V600E mutation—positive metastatic melanoma [9, 10]. The confirmed overall response rate was 53%. Most objective responses were noted as early as 6 weeks after the initiation of therapy, while the median duration of response was 6.7 months. Additional support was provided by Chapman et al. for the (BRIM-3) study group [11]. This phase III clinical trial demonstrated that vemurafenib produced improved rates of overall and progression-free survival in patients with previously untreated melanoma with BRAF V600E mutation. On the basis of those trials vemurafenib was approved by the FDA for the management of unresectable or metastatic melanoma [5–8], but its role as a neoadjuvant therapy has not been determined.

In patients with a solitary melanoma metastasis (especially brain metastasis), complete surgical resection appears to prolong survival [16–18]. However, whether patients are candidates for surgery—not all are—depends on several parameters, one of which is metastases size. In this report, we present a patient with melanoma metastatic to the axillary lymph nodes and to the brain who was treated with vemurafenib. One month of vemurafenib treatment was followed by successful surgical excision and, as revealed in the subsequent pathological analysis, was sufficient to induce complete tumor necrosis.

A review of the literature turned up three similar cases. In 2012 Fadaki et al. [19] reported on a patient with a bulky stage IIIC melanoma involving the left axilla and neck who received vemurafenib as neoadjuvant treatment. Tumor size decreased by more than 50%, which enabled the surgeons to perform a left modified radical neck and axillary dissection. Koers et al. [20] reported on a patient with axillary lymph node metastasis that was eligible for surgery after four 28-day cycles of vemurafenib treatment. The third case [21] was of a patient with a single melanoma brain metastasis that, 37 days after starting treatment with vemurafenib, decreased in size to an extent that precluded treatment with stereotactic radiosurgery (SRS). Postoperative MRI confirmed complete resection of the mass with no residual tumor evidence. Pathological analysis revealed extensive tumor necrosis.

To our knowledge, this is the first documented case in which vemurafenib induced complete tumor necrosis of both lymph node and brain metastases within one month or less, which indicated that the patient was a good candidate for excisional surgery.

The impressive effect of vemurafenib on the brain lesion is a clear demonstration that the drug can cross the blood brain barrier and efficaciously treat brain metastasis. This finding reinforces a prior report [22] of a 16-year-old girl whose brain metastasis was substantially reduced after treatment with vemurafenib.

In conclusion, vemurafenib may be an effective neoadjuvant therapy in metastatic melanoma. Treatment with the drug improves the conditions of patients with extensive, unresectable disease, making them good candidates for radical tumor resection in cases of bulky melanoma when primary resection is not feasible. The case presented here supports this approach and dictates the need for a larger study series that will exploit a promising new treatment for a disease which was so far inaccessible for surgical therapy.
